# Data From a Tertiary Care Center Reveal the Prevalence of Hepatitis B Among the Visiting Population

**DOI:** 10.7759/cureus.102675

**Published:** 2026-01-30

**Authors:** Sanjay K Sarkar, Samrat Biswas, Mihirjyoti Pathak, Sonuwara Begum, Kaushik Das, Syed Tanwir Alam

**Affiliations:** 1 Department of Microbiology, Tezpur Medical College and Hospital, Tezpur, IND; 2 Viral Research and Diagnostic Laboratory, Department of Microbiology, Tezpur Medical College and Hospital, Tezpur, IND; 3 Department of Microbiology, Pragjyotishpur Medical College and Hospital, Guwahati, IND

**Keywords:** hepatitis b virus, prevalence, rt-pcr, symptom assessment, viral load

## Abstract

Introduction: Like a silent contagion, the hepatitis B virus (HBV), if it remains undiagnosed, may subsequently lead to the development of chronic liver disease. The HBV spread is often observed in various ethnicities and can also be related to the socioeconomic status and habits of people. Our subject is a population of tribes and non-tribesmen belonging to the northern plains of the Brahmaputra valley to the foothills of Arunachal Pradesh, India, and therefore, the study of the spread of HBV in this area can be an interesting case in itself. This study aims to assess the prevalence of HBV retrospectively and to describe the epidemiology of disease symptoms.

Methodology: The selected population ranged from 0 to >80 years and included patients who attended outpatient departments or wards with suspected HBV infection, presenting with six disease-related symptoms. Serum samples were subjected to an enzyme-linked immunosorbent assay (ELISA) for preliminary screening, followed by reverse transcription-polymerase chain reaction (RT-PCR) to estimate viral load among positive cases. Statistical analysis and a prevalence study were conducted to correlate physical symptoms with HBV positivity and ethnic diversity.

Results: ELISA and subsequent RT-PCR analysis confirmed 165 positive cases. The positivity rates among males and females were 62.19% and 37.80%, respectively, with the highest proportion of cases originating from Sonitpur district, Assam. The most affected age groups were 21-40 and 41-60 years. Positive likelihood ratio analysis (baseline 2000 IU/mL) showed that diarrhea, pain, and vomiting were strongly associated with higher viral loads.

Conclusions: Data on HBV cases across different ethnic groups revealed seasonal spikes from mid-spring to summer, with male predominance in the 21-60 age range. Diarrhea, dysentery, and abdominal pain were common symptoms, affecting both tribal and non-tribal populations equally. Although most cases are serologically positive irrespective of viral load, studies tracking associated prevalent symptoms and likelihood ratios may help identify predictive therapeutic approaches to prevent HBV infection from becoming epidemic.

## Introduction

Hepatitis B virus (HBV) is an enveloped DNA virus belonging to the family Hepadnaviridae [1]. Detection and quantification of circulating HBV DNA in blood play as diagnostic role in identifying HBV infection in the host [2]. Clinically, HBV DNA titers vary greatly, from levels as high as 1010 copies/mL during acute HBV infection [3] to very low levels in patients undergoing antiviral therapy and in those with occult HBV infection [4]. However, commercial kits give a range of standards of copy numbers, 101 to 106 of HBV particles, which could be converted to an international unit of viral load, viz., 1 IU/mL, for the use of clinicians.

Related to this, real-time polymerase chain reaction (PCR) technology used for HBV DNA quantification assays offers high sensitivity and the broadest linear dynamic range [5-7]. Due to this high sensitivity, HBV DNA can be detected early during infection, preceding the appearance of other serological markers, such as HBsAg or anti-HBc [8].

As a consequence, testing for HBV DNA has emerged as a primary approach in the diagnosis and management of HBV infection. HBV DNA testing has now become routinely used in blood product screening (nucleic acid testing) and monitoring of patients with HBV during treatment. The National Guidelines for Diagnosis and Management of Viral Hepatitis (India) [9] suggest that patients with an HBV DNA load greater than 2,000 IU/mL should be categorized as “defer treatment and monitor.” If the patient is already HBsAg positive or negative by serological testing, they may be considered for antiviral therapy or other medications if abnormal biochemical changes are detected [10]. However, if viral load estimation is not considered a decisive co-factor, a positive biochemical test alone may not indicate the presence of HBV infection. In such cases, a solid plan to investigate suspected acute or chronic HBV infection cannot be reliably initiated. Therefore, interpreting viral load data alongside other clinical tests is crucial for guiding patient management, even when values are below the standard cut-off. It should be noted that the presence of HBV DNA in a sample can be confirmed by clinics and manufacturers for levels up to 200 IU/mL.

The North-Eastern region of India is home to the country’s most heterogeneous population, comprising more than 220 different ethnic groups [11]. Data on HBV prevalence and its detailed epidemiology in this region are limited. Assam, which hosts a diverse population including 12% of the tribal population of all North-Eastern states, shares interlinked borders with other states in the region and may exhibit distinct genotypic divergence and evolutionary patterns of the virus.

In this study, we aimed to observe the prevalence of HBV infection among the population visiting Tezpur Medical College and Hospital, specifically patients who were recommended for real-time PCR analysis to confirm the presence of Hepatitis B viral DNA in their samples. Simultaneously, viral load was estimated, fulfilling the clinician’s recommendations and guiding further treatment for the suspected patients. This retrospective analysis and the inferences drawn may provide insights into the prognosis of this serious disease across populations of different ethnicities, genders, and ages, as well as the potential risk of HBV spread in relation to viral load among the subjects.

## Materials and methods

The study was conducted at the Viral Research and Diagnostic Laboratory (VRDL) in the Department of Microbiology, Tezpur Medical College and Hospital (TMCH) during the period December 2021 to November 2023. The selected population ranged from 0 to over 80 years and included patients attending outpatient departments (OPDs) or wards. A suspected case of HBV was defined as a patient presenting with diarrhea and dysentery, jaundice, abdominal pain, unexplained or asymptomatic fever, vomiting, or other asymptomatic signs suggestive of hepatic disease, in accordance with the case report forms provided by the National Institute of Epidemiology, Chennai (NIE, ICMR). As the samples were prescribed by hospital clinicians for viral load estimation for routine diagnostic and treatment purposes, no separate patient consent was required. The costs of kits and consumables were covered by the National Viral Hepatitis Control Program and VRDL funds. This study was approved by the institutional ethics committee (vide SMET/TMC/Letter/99/2013/4821).

The hospital visiting population, after screening by a rapid card test for HBV antigen, was subjected to HBsAg ELISA testing following the manufacturer’s protocol (SD Biosensor, Suwon-si, Republic of Korea). Serum from positive samples was stored at -20 °C for further testing.

Viral DNA was extracted using the Hipura All Blood DNA Purification Kit (Himedia, India) via the spin-column extraction method, and purity was assessed using the Multiskan Sky reader (Thermo Fisher Scientific, Waltham, MA) equipped with a µ-drop plate [12]. RT-PCR was performed using the Qiagen artus RG-HBV kit on an ABI QuantStudio 5.0 platform. TaqMan chemistry was employed, with the FAM and JOE channels selected for detection of the HBV amplicon and the internal control, respectively. No passive reference dye was required. Viral load was quantified from a standard curve using five standards ranging from 1 × 10⁵ to 1 × 10¹ IU/µL. According to the manufacturer, 1 IU/mL is equivalent to 8.21 copies/mL of viral particles. The Ct value for positive controls was ≤36 for the respective HBV genome amplicons and was thus considered the in-house positive cut-off for discrimination.

The results were statistically analyzed using software and platforms such as MS Excel, Origin 8.6, online Python compilers, and Matplotlib. The average prevalence of symptoms among positive cases was calculated according to Spronk et al. [13] as: Average prevalence = ∑ (Percentage of affected population in each of five communities)/5. Likelihood ratio analysis was performed to assess the association of disease symptoms with actual viral load in positive samples [14]. A decision tree was also constructed to identify the strongest predictor among the five symptoms associated with positivity based on RT-PCR results.

## Results

Preliminary screening and RT-PCR analysis

The HBsAg ELISA conducted on patients visiting the TMCH OPD identified 195 patients with positive serum results, of whom 86% were from rural areas and 14% from urban areas. RT-PCR analysis confirmed positive Ct values in 165 patients.

Distribution of HBV cases among communities, areas, ages, and genders

**Figure 1:**
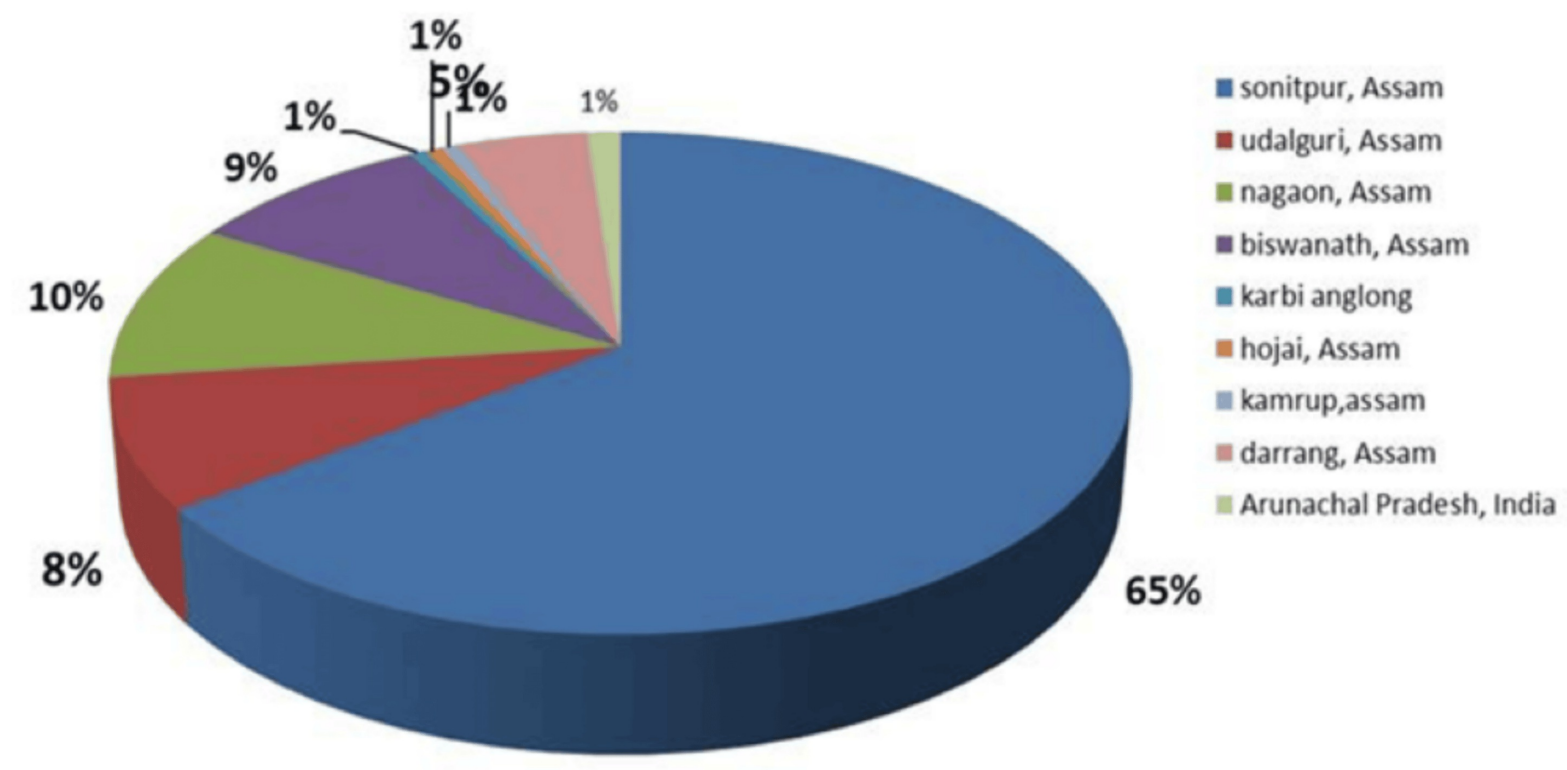
HBV-positive patient distribution within different districts. HBV, Hepatitis B virus

**Figure 2:**
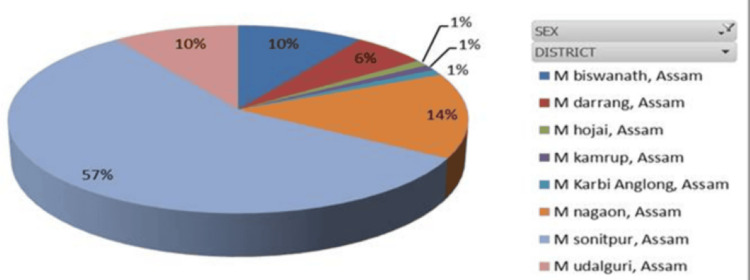
HBV-positive patient distribution among male patients from different districts. HBV, Hepatitis B virus

**Figure 3:**
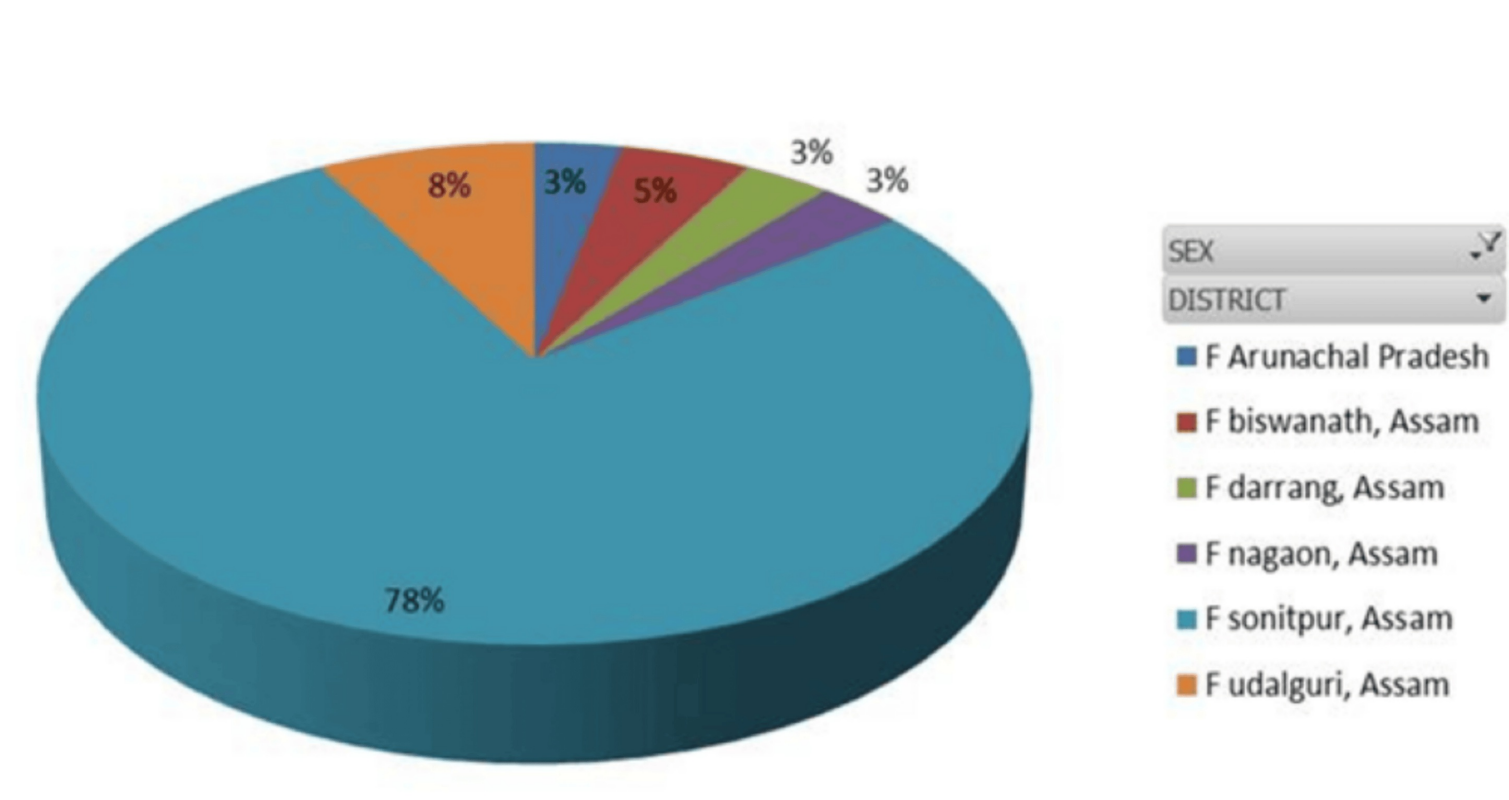
HBV-positive patient distribution among female patients from different social communities. HBV, Hepatitis B virus

RT-PCR analysis revealed that 62.19% of the positive patients were male and 37.80% were female. The patients came from several communities (Tribe, Non‑Tribe, Tea Tribe, Nepali, Unknown), with the following gender distribution: males, 25%, 37.5%, 12.5%, 17.5%, and 7.5%; females, 21.1%, 36.8%, 15.8%, 15.8%, and 10.5%, respectively. Most HBV-positive patients were from Sonitpur (64.6%), with others from Nagaon, Biswanath, Udalguri, Darrang, and Arunachal Pradesh. Age-group positivity in 2022 was 2.0% (0-10), 18.4% (11-20), 40.8% (21-40), 30.6% (41-60), and 8.2% (61-80), with the highest incidence between January and August occurring in the 11-60 age range. In 2023, the highest rates were observed in the 21-40 and 41-60 age groups (January to June: 41 and 32 cases, respectively). The male population in the age groups 0-10, 11-20, 21-40, 41-60, 61-80, and 81-100 years represented 33.3%, 68.75%, 53.75%, 72%, 73.33%, and 0%, respectively, while the female population represented 66.6%, 31.25%, 46.25%, 28%, 26.6%, and 0% of the total population. These data were further divided across the two years. Sex distribution varied by age: males predominated overall in the 11-80 age range, whereas in 2022, females were in the majority in the 21-40 age group (60.5%); distributions in 2023 were more balanced. No patients met the criteria in the 81-100 age group, which was therefore excluded from later calculations. Chi-square tests by age and sex for 2022 and 2023 were non-significant (*P* = 0.17 and 0.34), with t-tests yielding similar results (Figures 1-3).

**Figure 4:**
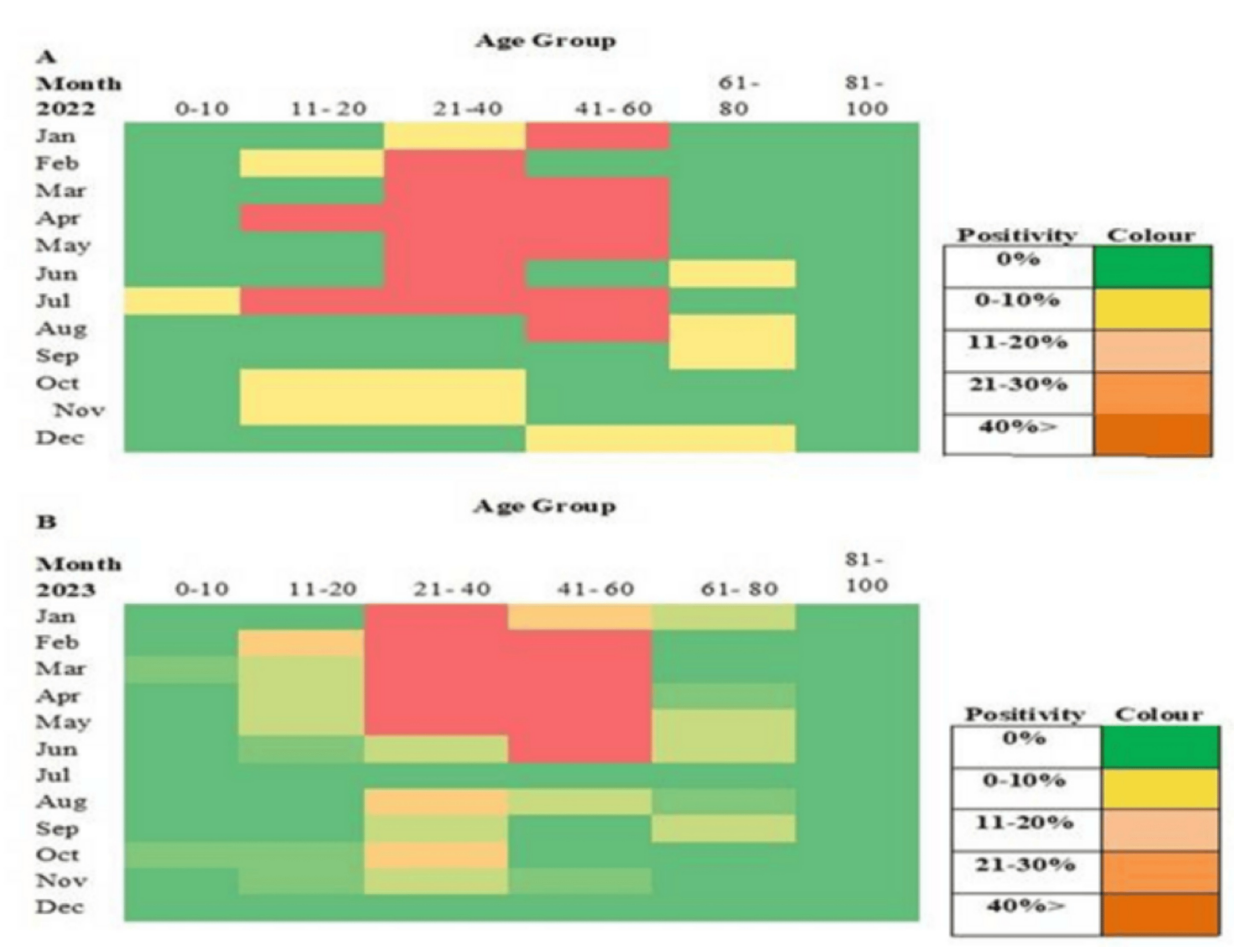
(A) and (B) Color heat-map analysis of HBV positivity percentages across different age groups throughout the months of 2022 and 2023. HBV, Hepatitis B virus

The age distribution of HBV-positive patients revealed district-wise variation. In Arunachal Pradesh and Hojai, all cases occurred in the 21-40 age group, whereas Kamrup and Karbi Anglong recorded all cases in the 41-60 age group. Biswanath exhibited a wider spread (11-80 years), with the majority (53.84%) in the 21-40 age group. Darrang showed 50% of cases in 21-40, while Sonitpur, the highest contributor, had cases across all age groups, peaking at 50% in 21-40 and 26.41% in 41-60. Nagaon and Udalguri also displayed broad age distributions, with predominance in the 21-60 age range. Heat map analysis provided additional clarity on the age distribution data (Figure 4).

Symptoms prevalence study and likelihood analysis

**Figure 5:**
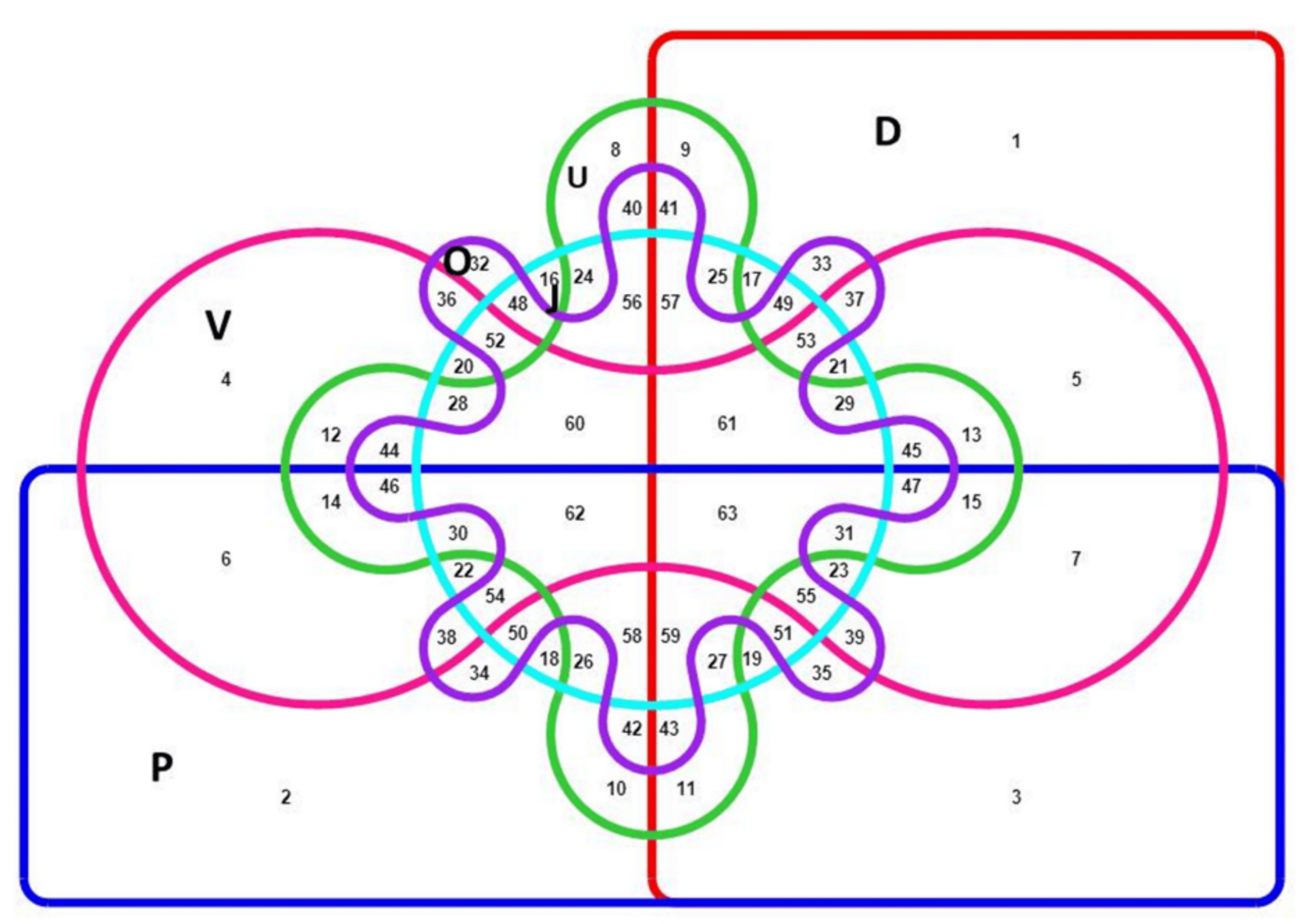
Venn diagram showing the distribution of symptoms among HBV RT-PCR-positive patients. The boxes labeled D, V, J, P, U, and O indicate diarrhea/dysentery, vomiting, jaundice, abdominal pain, unknown fever, and other symptoms, respectively. HBV, Hepatitis B virus; RT-PCR, real-time polymerase chain reaction

Venn diagram analysis demonstrated both isolated and coexisting symptoms, with diarrhea/dysentery, pain, and vomiting forming the most frequent combinations. The highest clustering was observed for diarrhea/dysentery, pain, and vomiting in this analysis (Figure 5).

**Figure 6:**
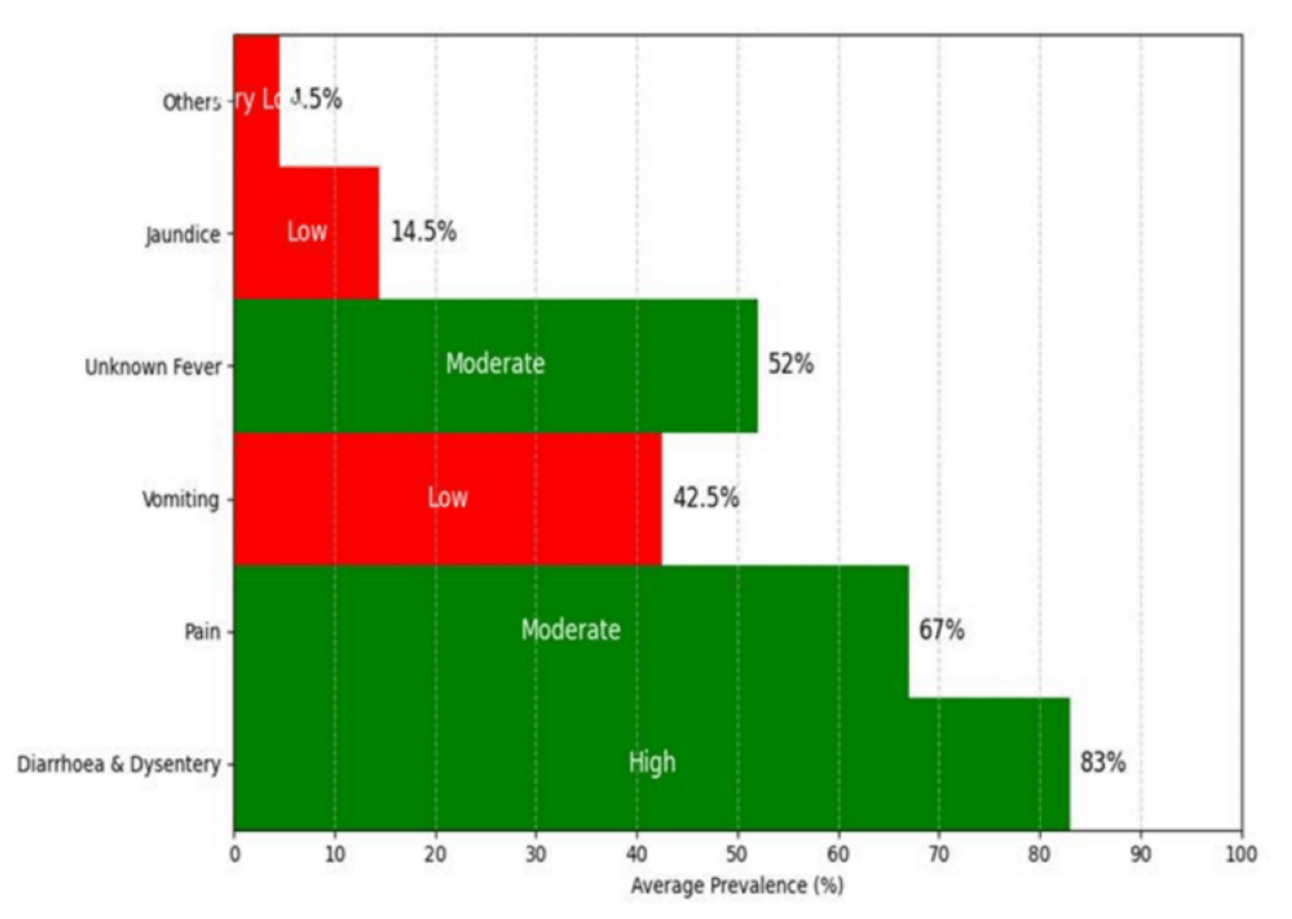
Graph showing the prevalence of different disease symptoms categorized as high, moderate, and low among the HBV-positive population. HBV, Hepatitis B virus

Symptom prevalence was as follows: diarrhea/dysentery (83%), abdominal pain (67%), vomiting (42.5%), unknown fever (52%), jaundice (14.5%), and others (4.5%) (Table 1, Figure 6). To examine the variation and distribution of these six symptom categories among all patients, counts were recorded by gender as follows. For males: 66, 39, 35, 2, 27, 7; for females: 41, 40, 19, 4, 17, 5. An initial Chi-square test indicated no significant association between gender and disease symptoms (*P* > 0.05).

**Table 1:** Percentage of disease impact on each community and symptom prevalence. Chi-square statistic: 48.88; degrees of freedom: 20; *P*-value: 0.00032.

Disease symptoms	Effected percentage (%) in different communities					
	Non-tribe	Tribe	Tea tribe	Nepali	Unknown	Symptom prevalence among positive cases
Diarrhea and dysentery	75%	100%	100%	90%	50%	83%
Pain in the abdomen	25%	100%	100%	60%	50%	67%
Vomiting	12.5%	100%	50%	50%	0.0%	42.5%
Unknown fever	50%	100%	100%	10%	0.0%	52%
Jaundice	12.5%	0.0%	0.0%	10%	50%	14.5%
Others	12.5%	0.0%	0.0%	10%	0.0%	4.5%

**Figure 7:**
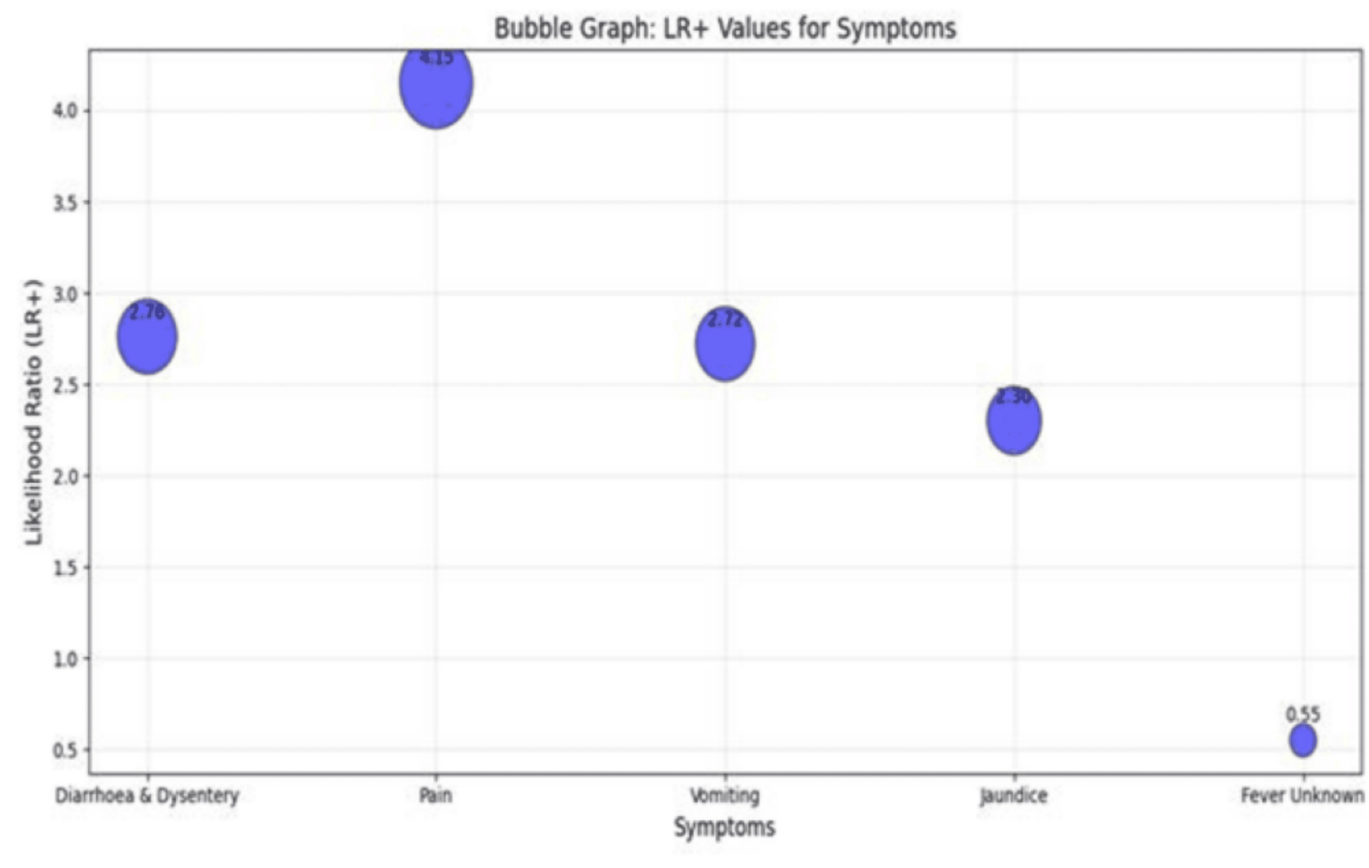
Bubble graph showing the distribution of LR+ of viral load against diarrhea and dysentery, abdominal pain, vomiting, jaundice, unknown fever, and other symptoms, with an association to values where >2,000 IU/mL was set as the baseline.

The likelihood ratio was calculated to assess the association of diarrhea/dysentery, abdominal pain, vomiting, jaundice, unknown fever, and other symptoms with viral load (IU/mL). The results were as follows: for LR ≥ 2000, the values were 6, 5, 0, 0, 0; for LR 1,000-2,000, they were 6.11, 2.78, 0, 0, 0; and for LR 200-1000, they were 6.25, 3.75, 0, 0, 0, respectively. In this analysis, >200 IU/mL was used as the baseline and counted as 1. If the baseline were set at >2000 IU/mL, consistent with the World Health Organization (WHO) and the National Viral Hepatitis Control Program (NVHCP) standards for defining an RT-PCR-positive case, the six symptom categories showed LR+ results of 2.7, 4.15, 2.72, 2.30, 0.55, 0.25, and N/A, respectively (Figure 7). These findings suggest symptom clustering in younger and middle-aged groups, with diarrhea/dysentery and abdominal pain serving as potential clinical correlates of high HBV viral load.

## Discussion

Assam is a land of diversity, not from the angle of flora and fauna, but from the variety of ethnicities that have settled in the Brahmaputra and Barak valleys for thousands of years. It is also a hub for different traveller ethnic groups, communities, and permanent settlers, who have finally built the mixed population of tribes and non-tribes dwelling in the hills and plains of this amazing state of India. Tezpur (Sonitpur district), bordering Arunachal Pradesh - a gateway to the Himalaya, suspected to have a rise in HBV positivity in the population [15], was selected for this investigation. Among 195 patients screened at TMCH, 165 were confirmed HBV-positive via RT-PCR. Male prevalence (62.2%) exceeded female (37.8%) (ratio 1:0.6). Community-wise, non-tribes contributed the highest share, but Chi-square analysis did not show significant sex-based differences across communities (*P* = 0.85).

Demographically, 86% of positives arose from rural areas, consistent with Sonitpur’s population profile [16]. It was found that in 2022, positivity peaked in June and July among the 21-40-year-old population, while 2023 showed a higher overall occurrence (46.08%), especially from May to July. Analysis of variance (ANOVA) confirmed significant inter-year variation (*P* = 0.0043, *F* = 8.45). Tribal populations showed higher infection, consistent with earlier reports [17]. Gender differences in HBV epidemiology are well-documented, with males more affected [18], a pattern also seen in China, which is geographically near our study area. It was observed that patients from all selected areas participated, ranging from the 11-20 to 41-60 age groups, but Sonitpur district showed 100%, 75%, 66.25%, 56%, and 67% of the population (0-10, 11-20, 21-40, 41-60, and 61-80 age groups), indicating that the majority of HBV-affected patients came from their home districts. From the perspective of patient numbers, it was clearly observed that the 21-40 and 41-60 age groups were the most affected within the entire visiting area.

The burden of HBV is a significant public health problem in India despite an effective vaccine. In a study conducted by a virology laboratory in Uttar Pradesh, India, a higher seroprevalence was observed in the age group (16-30 years), which contributed half of the total prevalence in that study [18-19]. Rokade et al. have reported an increasing prevalence among the 15-45 year age group and found that 51.9% of individuals were HBV positive [20]. This analysis indicates the variance of interest or awareness of HBV among the general population and the necessity to vaccinate the young generation, as it is seen that this group is often prone to hepatitis infection. External symptoms were often found in HBV-infected persons throughout the development of the disease, and in our case, we have also stratified the patient information into these six major types. Among HBV-positive individuals aged 21-40, there is a strong correlation between HBV positivity and the manifestation of symptoms. This age group is most symptomatic, with a substantial proportion experiencing gastrointestinal symptoms (such as diarrhea and abdominal pain) and liver-related issues (such as jaundice) [21]. Although studies showed that even the 0-10-year age group was diagnosed as HBV positive, such an occurrence is not very common [22,23].

Symptom analysis revealed diarrhea/dysentery (83%) and abdominal pain (67%) as dominant, followed by unknown fever (52%), vomiting (42.5%), and jaundice (14.5%). Venn diagram analysis showed frequent co-occurrence, particularly diarrhea with pain (47.5%). Heatmaps confirmed that these symptoms clustered in the 21-40 and 41-60 age groups. Interestingly, as a whole, the tribal people, which include both tribe and tea-tribe communities, show almost half of the studied population (50% for males and 52.6% for females). These two key disease symptoms, along with vomiting and unknown symptomatic fever, showed 100% presence when later diagnosed positive by PCR. These people mostly dwell in rural areas of the study sites and are known for their social habits of alcohol consumption, smoked food consumption, body piercing, and tattooing. India has a large tribal population residing in different pockets across the country, and prevalence is very high, sometimes reaching 65%, including an occult infection rate of 10-9.5% [24]. Assam, as a tribal-majority state, cannot be indifferent.

**Figure 8:**
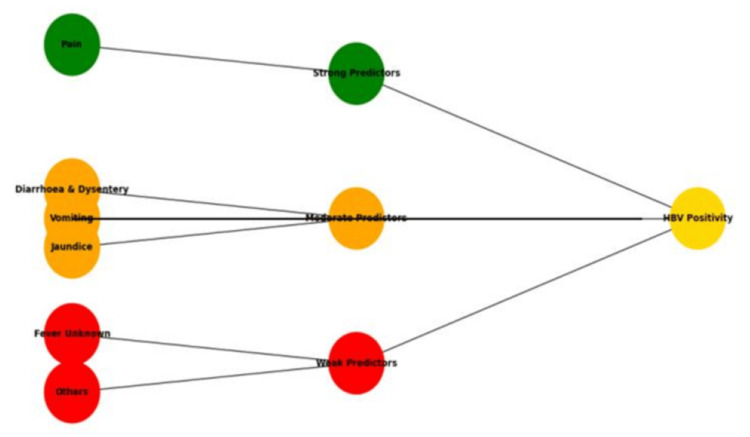
Development of a predictive tree on the basis of LR+ values corresponding to baseline >2000 IU/mL of viral load in each positive case with symptoms. Dominance of 60% was hypothetically considered a strong predictor, 45%-59% as a moderate predictor, and less than 45% as a weak predictor output in the tree construction. HBV, Hepatitis B virus

Positive Likelihood Ratio analysis (baseline 2000 IU/ml) showed that diarrhoea, pain, and vomiting were strongly associated with higher viral loads [25,26]. The LR+ results were 2.76, 4.15, and 2.72, respectively, for these symptoms, and since they were above 1.0, this indicated a strong and positive association between HBV positivity and the presence of these disease symptoms [27]. Hence, following this theory, a patient’s positivity, which can be considered for critical treatment, can be determined by understanding the impact of these selected symptoms associated with HBV prognosis. The LR+ values for diarrhea and dysentery, pain in the abdomen, and vomiting can likely be related to the positivity of a sample quantified in the form of viral loads and can be considered a predictive key for developing a treatment policy, as shown in a predictive tree (Figure 8). Overall, the HBV burden in Assam is concentrated in rural, young-to-middle-aged males, particularly tribal groups, with gastrointestinal symptoms predictive of higher viral replication.

## Conclusions

Any pattern of spread of an infectious disease can only be understood when a studied population is assessed with different parameters, over time, and with consideration of a baseline measurement. Our study, conducted post-COVID on suspected HBV cases across ethnic groups in Sonitpur, Assam, revealed seasonal spikes from mid-spring to summer, with male predominance in the 21-60 age range. Diarrhoea, dysentery, and abdominal pain were common symptoms, affecting both tribal and non-tribal populations equally. Despite low viral loads on RT-PCR, most patients were serologically positive, suggesting delayed hospital visits and limited vaccine awareness. Our study continues to identify more predictive parameters to understand disease pathogenesis and pre- and post-vaccination changes with more advanced diagnostic strategies.
